# Tubercular Hepatic Abscess: An Incidental Finding

**DOI:** 10.7759/cureus.35447

**Published:** 2023-02-25

**Authors:** Swati Dhungel, Satya Mishra

**Affiliations:** 1 Internal Medicine, John H. Stroger, Jr. Hospital of Cook County, Chicago, USA; 2 Gastroenterology, John H. Stroger, Jr. Hospital of Cook County, Chicago, USA

**Keywords:** liver abscess aspiration, extrapulmonary tuberculosis (eptb), isoniazid resistance, tubercular abscess, hepatic abscess

## Abstract

Tuberculosis (TB) is an infectious disease caused by a bacterium, *Mycobacterium tuberculosis*. It is known to generally affects the lungs, but it can also affect multiple other parts of the body. Liver involvement with hepatic abscess is an infrequent manifestation of TB which is missed because of the rarity and non-specific symptoms, especially in the west. A thorough literature review shows very few case reports published in the western world. We present a rare case of isoniazid-resistant pulmonary TB associated with a hepatic abscess in the United States. It was diagnosed by aspiration of the abscess that later grew *M. tuberculosis* and treated with antitubercular drugs.

## Introduction

A hepatic abscess is a rare manifestation of tuberculosis (TB) whether it be an isolated primary hepatic tuberculous abscess or associated with pulmonary TB. The diagnosis is missed because of the rarity and non-specific symptoms that include fever, right upper quadrant pain, and anorexia. Hepatomegaly is the most common physical finding and jaundice is uncommon [1]. Diagnostic modalities like ultrasound and computed tomography (CT) can also miss it. The gold standard for diagnosis is the demonstration of an acid-fast *Mycobacterium *in aspirated pus [2].

Hepatic TB has been reported in 10-15% of patients having extrapulmonary TB, but tubercular liver abscess is extremely rare with a prevalence of 0.34% [1]. There have been only a few case reports with this condition even in TB-prevalent regions. We present a rare case of isoniazid-resistant pulmonary TB associated with a hepatic abscess in the United States.

## Case presentation

A male in his late teens with no prior medical history presented to the Emergency Department after two months of sore throat and dry cough that had been gradually getting worse for the past few days. It was associated with subjective fevers, chills, and nonspecific weight loss. There were no reported night sweats or masses anywhere in the body. Of note, his roommate tested positive for isoniazid-resistant TB the same week.

On initial evaluation, vitals were unremarkable with a blood pressure of 108/59mmHg, heart rate of 94 beats per minute, a temperature of 36.8 degrees Celsius, and oxygen saturation of 98% in room air. Physical examination revealed a thinly built male coughing relentlessly, with decreased air entry in bilateral lower lung fields and inspiratory wheezing in the right upper lung field. Cervical spine tenderness was also present.

Laboratory tests done are listed in Table 1, Table 2, and Table 3. The normal values given below are in the range as standardized by the hospital lab where the tests were done.

**Table 1:** Complete metabolic panel This table shows that the complete metabolic panel of the patient was within normal limits. BUN: blood urea nitrogen

	Na	K	Cl	CO2	BUN	Cr	Ca	Phos	Mg	Uric Acid	Total Protein	Albumin
Unit	mEq/L	mEq/L	mEq/L	mEq/L	mg/dL	mg/dL	mg/dL	mg/dL	mg/dL	mg/dL	g/dL	g/dL
Normal	135-145	3.5-5	100-110	23-31	8-20	0.6-1.4	8.5-10.5	2.5-4.5	1.8-2.7	3-7	6.4-8.3	3.8-5.2
Observed	137	4.4	98	27	9	0.8	8.2	3.5	2.1	4.5	7	3.6

**Table 2:** Hepatic profile The hepatic profile of the patient showed deranged liver enzymes. ALP: alkaline phosphatase; GGT: gamma-glutamyl transferase; AST: aspartate aminotransferase; ALT: alanine aminotransferase; LDH: lactate dehydrogenase

	Total Bilirubin	Direct Bilirubin	ALP	GGT	AST	ALT	LDH
Unit	mg/dL	mg/dL	U/l	U/l	U/l	U/l	U/l
Normal	0.2-1.2	0-0.2	20-120	3-60	0-40	5-35	85-210
Observed	0.5	0.1	147	247	47	43	246

**Table 3:** Complete blood count The complete blood count shows anemia and thrombocytosis which explain underlying chronic inflammation. WBC: white blood cells; RBC: red blood cells; Hb: hemoglobin

	WBC	RBC	Hb	Platelets
Unit	k/uL	mil/uL	g/dL	k/uL
Normal	4.4-10.6	4.15-5.5	12.9-16.8	167-369
Observed	10	4.37	11.9	630

Other investigations like HIV screen, COVID screen, Blastomyces antigen, and Hepatitis panel were all negative.

Chest X-ray showed consolidative opacity in the right upper lung lobe and hazy airspace opacity in the right lower lung lobe with small right pleural effusion which was concerning for active TB.

QuantiFERON tuberculosis came negative, but the *Mycobacterium tuberculosis* polymerase chain reaction (PCR) of the sputum was positive. Acid-fast bacilli (AFB) smear and *Mycobacterium* species culture of the sputum were sent, which later resulted in positive. Antitubercular therapy started with levofloxacin 500 mg, ethambutol 1200 mg, pyrazinamide 1500 mg, and rifampin 600 mg given exposure to isoniazid-resistant strain.

**Figure 1:**
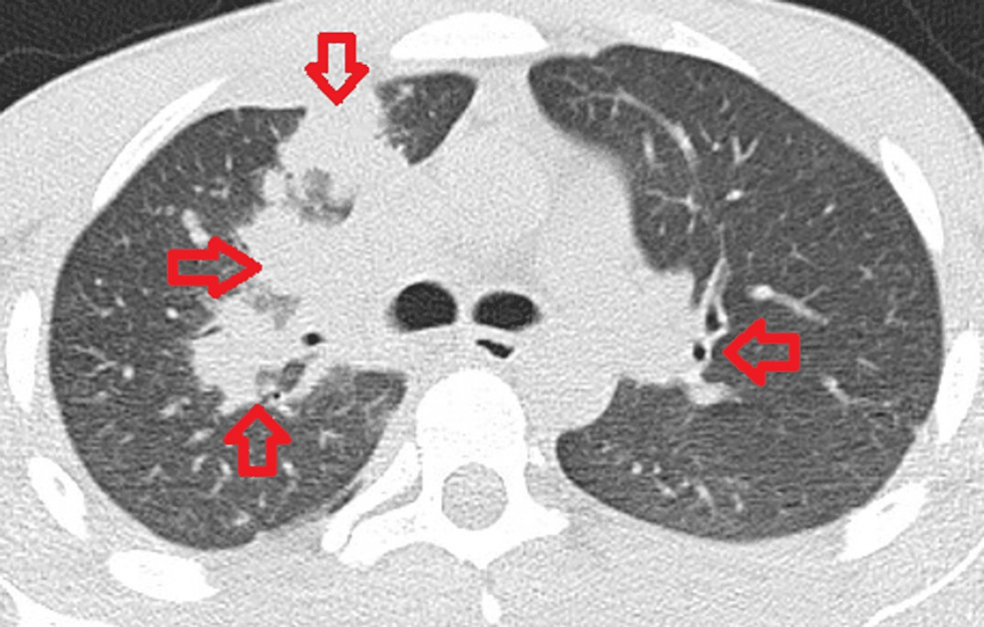
Right upper lobe opacity with bilateral lung nodules (finding shown by red arrows)

CT chest was done to better characterize the chest X-ray finding. It showed a large right upper lung cavitary mass with additional bilateral pulmonary nodules and masses, nodularity of the right pleura, and adjacent subcapsular lobulated lesions noted in the right hepatic lobe. The lung finding has been shown in Figure 1.

Magnetic resonance imaging (MRI) spine showed enhancing soft tissue centered along the anterior margin of the C4 vertebral body with loss of anterior cortex and mild marrow enhancement with sparing of disc spaces, consistent with Pott's disease.

**Figure 2:**
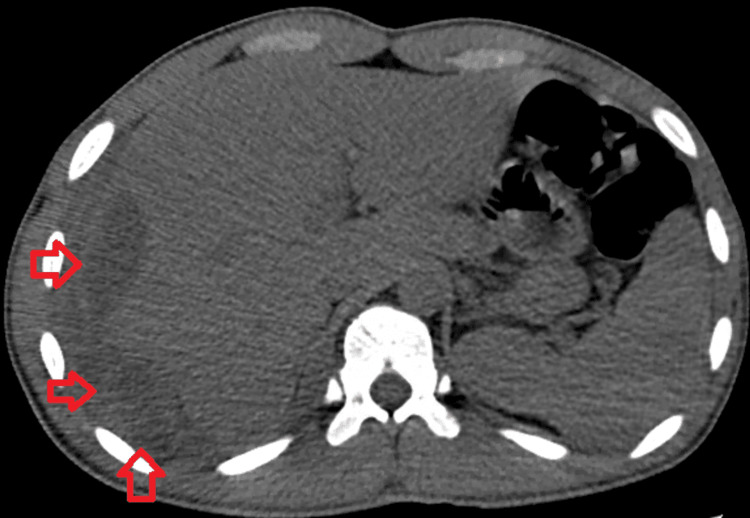
Multiple liver abscesses shown with red arrows

Given the elevated liver enzymes and incidental liver lesions on chest CT, a dedicated CT abdomen pelvis was ordered. This showed right pleural thickening extending inferiorly to the diaphragm and posterior and lateral aspect of the right liver capsule causing a mass effect in the lateral and posterior aspect of the right upper lobe. The liver was enlarged with a measured length of 18.2 cm. The liver finding has been shown in Figure 2.

CT-guided aspiration of the liver abscess was done that drained 30cc of purulent malodorous fluid. The bacterial gram stain and culture of fluid were negative. Mycobacterium PCR of the fluid was also negative, but the AFB smear and *Mycobacterium *species culture of the fluid came back positive that confirmed the liver abscess was tubercular.

The patient was discharged after a repeat sputum AFB was negative with antitubercular treatment to be continued for 9-12 months as per the Canadian Thoracic Society [3] for pulmonary TB with liver abscess and Pott's spine.

## Discussion

Hepatic TB is a rare form of extrapulmonary TB that is reported in 10-15% of patients having extrapulmonary TB [1]. The various forms of liver involvement by TB are (i) diffuse involvement with miliary or pulmonary TB; (ii) diffuse involvement without evidence of existing TB in any organ; (iii) focal or nodular lesions in the liver, multiple or solitary and present as tuberculoma or abscess [2]. Hepatic TB can also be classified as (i) miliary TB, (ii) primary pulmonary TB with lung involvement, (iii) primary hepatic TB, (iv) tuberculoma, and (v) tubercular cholangitis [4].

Tubercular liver abscess is extremely rare with a prevalence of 0.34% [5]. Our case falls under primary pulmonary TB with lung involvement and the involvement of the liver is focal to abscess formation.

Symptoms of tuberculous liver abscess are nonspecific that include vague abdominal pain, fever, loss of appetite, and weight loss [6]. The most common physical finding is hepatomegaly and jaundice is uncommon [7]. Due to the nonspecific symptoms and the rare occurrence of the disease, it is usually missed.

Radiological diagnosis of the tuberculous liver abscess has a low specificity [8]. The hypodense focal lesions seen on CT can also be confused with amoebic or pyogenic liver abscesses and hepatoma. Hence the definite diagnosis is done by demonstration of AFB in pus, aspirate, or biopsy specimen. *M. tuberculosis* culture is the gold standard method for the detection of the bacteria but needs viable microorganisms and a long incubation period of around 6-8 weeks. For a rapid diagnosis, Mycobacterium TB PCR can be used to improve TB management [1].

Antitubercular therapy with percutaneous drainage of the pus is the preferred management [9] and the same was done in our case.

## Conclusions

*M. tuberculosis* can affect most of the organs in the body; the most common one being the lung. Any involvement including the lung is rare in the west and even if found is common among the immigrants and immunocompromised population. Liver involvement with hepatic abscess has been found only in a handful and can be confused with other common causes like bacterial liver abscesses and even malignancies. Through this article, we highlight the importance of considering TB as a cause of hepatic abscess, as early diagnosis prevents unnecessary medications and interventions in the patients which in turn reduces morbidity and mortality. Surgical intervention is not required unless the abscess is too large causing symptoms or does not resolve after antitubercular treatment.
